# Collapse, social tipping dynamics, and framing climate change

**DOI:** 10.1177/1470594X231196432

**Published:** 2023-08-21

**Authors:** Daniel Steel, Kian Mintz-Woo, C. Tyler DesRoches

**Affiliations:** 8166 University of British Columbia, Vancouver, British Columbia, Canada; 8795 University College Cork, Cork, Ireland; 31362 International Institute for Applied Systems Analysis, Laxenburg, Austria; Arizona State University, Arizona, USA

**Keywords:** societal collapse, climate change, climate ethics, climate justice, intergenerational justice, mitigation, tipping points

## Abstract

In this article, we claim that recent developments in climate science and renewable energy should prompt a reframing of debates surrounding climate change mitigation. Taken together, we argue that these developments suggest (1) global climate collapse in this century is a non-negligible risk, (2) mitigation offers substantial benefits to current generations, and (3) mitigation by some can generate social tipping dynamics that could ultimately make renewables cheaper than fossil fuels. We explain how these claims undermine familiar framings of climate change, wherein mitigation is understood as self-sacrifice that individuals and governments must be morally persuaded or incentivized to undertake.

## Introduction

In his speech to the 24^th^ Conference of Parties to the United Nations Framework on Climate Change in 2018, Sir David Attenborough warned, “If we don’t take action, the collapse of our civilizations and the extinction of much of the natural world is on the horizon.”^1^ Climate scientists have also made warnings about collapse risks. Kevin Anderson (2012: 29), referring to a projection of global mean temperature by 2100 given business as usual, writes, “there is a widespread view that a 4°C future is incompatible with any reasonable characterisation of an organized, equitable and civilized global community” (also cf. Ripple et al., 2021; Stern, 2022; Tollefson, 2021). Similar warnings have been made in writings aimed at the general public (Lynas, 2020; McKibben, 2019; Thunberg, 2019; Wallace-Wells, 2019) and have inspired an international wave of climate activism. And the Summary for Policymakers of the Intergovernmental Panel on Climate Change's (IPCC) Sixth Assessment Report (AR6) states with very high confidence that “There is a rapidly closing window of opportunity to secure a liveable and sustainable future for all” (IPCC, 2023). Let the term *climate collapse* refer to societal collapse caused by anthropogenic climate change. In this article, we explore the concept of climate collapse and suggest that, in conjunction with recent scientific and technological developments, it has significant implications for how the collective action problem raised by climate change mitigation should be framed.

First, we make the case that climate collapse should be taken seriously as a risk for current generations. Most experts believe that a + 4°C world poses severe risks to the stability of contemporary societies (Anderson, 2012; Bettis et al., 2017; World Bank, 2012), while high-emission socioeconomic pathways (formally, SSP3-7.0 and SSP5-8.5) in AR6 include that level of warming in the very likely (66%–100%) range for 2081 to 2100. The risk of collapse, therefore, is a real for those who may live to see the last two decades of this century. But it is also a significant concern for older people as well. Adults living in locations with high exposure to climate risks and limited resources for adaptation may experience the collapse of their own communities before 2080. And the risk of climate collapse can matter even for adults who are unlikely to experience it in their own lifetimes. Scheffler (2013, 2018) argues that belief in personal and social connections to a posthumous future is important to the well-being of most people. If this is right, then a serious risk of climate collapse is also a threat to older people who won’t live to see it.

However, not all of the news about climate change is bad. Recent research finds that climate change mitigation as represented by low-emission pathways from IPCC reports would produce a decrease in global heating in comparison to higher-emission scenarios *within two decades* from the beginning of a phase out of fossil fuels (Shindell and Smith, 2019). Thus, reduced global heating from mitigation, and consequently reduced climate collapse risks, would be felt within the lifetimes of current generations. A second hopeful line of climate science pertains to the diffusion and economics of renewable energy, such as photovoltaic solar and wind. The declining costs of these technologies have been due in large measure to policies promoting their development and uptake (Nemet, 2019). This means that mitigation policies in one place can accelerate social tipping dynamics that reduce costs of renewables and ultimately make them more economically attractive than continued fossil fuel use (Ilona Otto et al., 2020; Sharpe & Lenton, 2021; Winkelmann et al., 2022).

We claim that combining these frightening and hopeful scientific developments should prompt a reframing of collective decision-making about climate change mitigation. Debates about climate change are often framed around the idea that mitigation involves a self-sacrifice, either for countries in the global North that are less vulnerable to climate impacts (Posner and Wesibach, 2010), for current generations who won’t live to see benefits of mitigation (Gardiner, 2011), or for each person because mitigation, while collectively beneficial, is individually costly (Steele, 2021). Given these framings, key questions are how to persuade wealthier countries to mitigate, how to convince current people of their moral obligation to sacrifice for future generations, and how to deter free-riding. We claim that each of these framings of mitigation is no longer tenable.

The impending risk of climate collapse shows that climate change is not merely a concern for especially vulnerable countries: it is a risk that should concern everyone. And since aggressive mitigation is likely to have discernible climatic effects within two decades, it stands to significantly benefit current generations. Moreover, social tipping dynamics and declining prices of renewable energy undermine the idea that mitigation can be modeled as a prisoner's dilemma, where mitigation is against each individual's self-interest no matter what others do. Consequently, we propose a framing founded on the premises that the risk of climate collapse is non-negligible, current generations benefit from mitigation, and actions that push us past social tipping points can make renewables cheaper than fossil fuels. Given this framing, the key question is to find actions that most fairly and efficiently accelerate the transition.

The remainder of this article is as follows. The section “Clarifying the concept of climate collapse” clarifies the concept of climate collapse, drawing on related discussions from the philosophical literature. The section “Taking climate collapse seriously” discusses evidence suggesting that climate collapse is a non-negligible possibility, supporting the claim that, for many current people, mitigating climate change would lessen the likelihood of outcomes that they have substantial reason to avoid. In “And now for the good news”, we discuss the more hopeful developments in climate science and renewable energy technologies and discuss their implications for some familiar framings of climate change. The section “Reframing climate ethics” presents our alternative framing, and our “Conclusion” finishes with a reflection on framings of climate change that characterize mitigation as self-sacrifice.

## Clarifying the concept of climate collapse

Climate collapse is societal collapse caused by anthropogenic climate change. For the purposes of this discussion, we define *societal collapse* as a steep decline in a society's material and organizational capacities to perform essential functions such as providing food, housing, and security (Steel et al., 2022). Similar to the concept of collapse more generally, our definition consists of two key components: a variable that *declines* and a *threshold* that indicates a loss of basic functionality. For example, a fishery collapse implies not merely that fish stocks have diminished, but that they have been so reduced that the fishery is no longer a basis for livelihoods. The declining variable in our definition of societal collapse is the material and organizational capacity of the society. The threshold of basic functionality in our definition is the capacity to provide services and conditions needed for life and mutually beneficial social interactions. The term *capacity* is key here: it is not merely that the government has failed to maintain these conditions and can be replaced with more competent leadership that could. It is that the society lacks the organizational or material bases needed to do so. In the case of societal collapse, this loss of capacity is both pervasive, in the sense of adversely impacting the majority of the population, and difficult to reverse. These two conditions are intended to distinguish collapses from minor or short-lived adverse events.

Our definition suggests a straightforward reason why societal collapses can be catastrophic. Collapse as we understand it entails a loss of societal capacity to enact policies and plans, provide physical security, maintain functioning institutions, respond to crises, or secure basic needs, such as access to food or water. Since almost everyone depends for their survival on the material and organizational capacities of the societies in which they live, societal collapse stands to have severely adverse consequences for well-being.^2^

Our definition of societal collapse can be connected with climate collapse scenarios discussed in the philosophical literature. In *Climate Change and Future Justice*, McKinnon (2012: 60–61) writes:

The net result of a 5°C increase would be worldwide famine, severe conflict over water in the world's desertified regions, and extreme territorial insecurity for those living in the dwindling inhabitable areas as an archipelago of refuges is put under increasing pressure by hungry and desperate people.

McKinnon suggests that these conditions of extreme resource scarcity would “make the joint pursuit of justice impossible” because self-preservation would overwhelm social norms and any ideas of sacrificing for a greater social good (2012: 61). Although McKinnon does not use the word “collapse” in connection with societies, what she describes is naturally interpreted as climate collapse on a global scale.

Mulgan's (2014; 2018) “broken world” paints a somewhat less bleak picture. The broken world faces major economic and environmental scarcity as a result of climate change, large swathes of the planet have become uninhabitable, and many societies that currently exist have long since collapsed. On the brighter side, “modern largescale industrialised societies” still exist the broken world (Mulgan, 2018: 536; also cf. Oreskes and Conway, 2014). Mulgan imagines that these societies implement “survival lotteries” to ensure that population does not outstrip resources, wherein the government, when confronted by food or water shortages, culls the population according to a formula that is deemed fair (2018: 537). In Mulgan's broken world, then, climate collapse is widespread but not quite global, as some large, non-collapsed societies remain.

Wündisch (2019) discusses ethical obligations for wealthier nations created by territory loss due to climate change, which, by reducing a nation's access to needed resources, may undermine its capacity for political self-determination. According to Wündisch, “a people's continued political self-determination, in practice, usually relies upon strong institutions and infrastructure” (2019: 322). In our terms, Wündisch is concerned with the *local* climate collapses, and argues that these should be seen as moral wrongs in their own right and that wealthier nations consequently have an obligation not merely to accommodate refugees but also to relocate entire societies. Wündisch's discussion is significantly more optimistic than McKinnon's and Mulgan's scenarios insofar as he assumes that climate change does not threaten the capabilities of wealthier nations to compensate for climate loss and damage.

This selection of philosophical literature related to climate collapse draws attention to three types of scenarios: collapse across the entire globe, severe declines in social capacity globally along with widespread but not global collapse, and local collapses of certain vulnerable states combined with stability of wealthier, less vulnerable countries. For convenience, we can refer to these as *global collapse*, *the broken world*, and *local collapse*, respectively.^3^ We now consider reasons why climate collapse in these senses should be taken seriously.

## Taking climate collapse seriously

In this section, we provide reasons for thinking that the risk of climate collapse should be taken seriously given the science reviewed in the most recent set of IPCC assessment reports. However, we do not predict that climate collapse will occur, nor do we even attempt to assign a probability to that outcome. Instead, we claim that climate collapse is a non-negligible risk given our current knowledge.

Warnings that unchecked climate change may lead to societal collapse often cite +4°C as a crucial threshold at which this risk becomes a serious possibility (Anderson, 2012; Lynas, 2020). A World Bank report titled *Turn Down the Heat: Why a 4°C Warmer World Must be Avoided* characterizes a + 4°C world as one of “unprecedented heat waves, severe drought, and major floods in many regions, with serious impacts on human systems, ecosystems, and associated services” (2012: xiv). It discusses the likelihood of compounding climate impacts, and states that at 4°C warming “the risk of crossing critical social system thresholds will grow,” at which point, “institutions that would have supported adaptation actions would likely become much less effective or even collapse” (2012: xviii). Bettis et al. (2017) discuss concepts of societal collapse, identify “climate ruin” with a 4°C or greater increase in global mean temperatures from pre-industrial levels, and argue that the world is currently running a risk of climate ruin in excess of the risk of financial ruin allowed for insurance companies (also cf. Pal and Eltahir, 2016). Civilization destabilizing risks of a + 4°C world include some densely populated regions becoming uninhabitable due to sea level rise or extreme heat (Bolleter et al., 2021; Li et al., 2020; Hauer et al., 2020) and synchronous crop failures leading to global food shortages (Tigchelaar et al., 2018). In his book, *Our Final Warning*, Lynas (2020) sums up this grim picture: “With four degrees of heating, massive shocks to society will be taking place, threatening or even destroying modern industrial civilization because of mass starvation, flooding, and the loss of large areas of the tropics and sub-tropics to extreme heat and drought.”

There is, then, some basis for thinking that risk of global collapse is a serious concern if global heating reaches 4°C, which allows us to connect climate collapse and the IPCC's assessment reports. The most recent of these, the AR6, considers five shared socioeconomic pathways (SSPs), which can be divided into two high, one intermediate, and two low emission pathways. In the two high emission SSPs (SSP3-7.0 and SSP5-8.5), CO_2_ emissions increase at a rate similar to that of the past decade for most of the twenty-first century. In each high emission SSP, + 4°C falls within the “very likely” (i.e., 66% to 100%) range for 2081–2100 (see Table 1). Thus, if +4°C is taken as a proxy for risk of global collapse, the AR6 tells us that this risk is a non-negligible possibility given the IPCC's high emissions SSPs.

**Table 1.** IPCC's temperature projections for different scenarios (source: IPCC et al., 2021: table SPM.1, 14).

	Near term, 2021–2040		Mid-term, 2041–2060		Long term. 2081–2100	
Scenario	Best estimate (°C)	*Very likely* range (°C)	Best estimate (°C)	*Very likely* range (°C)	Best estimate (°C)	*Very likely* range (°C)
SSPI-1.9	I.5	1.2 to 1.7	1.6	1.2 to 2.0	1.4	1.0 to 1.8
SSP1-2.6	I.5	1.2 to 1.8	1.7	1.3 to 2.2	1.8	1.3 to 2.4
SSP2-4.5	I.5	1.2 to 1.8	2.0	1.6 to 2.5	2.7	2.1 to 3.5
SSP3-7.0	I.5	1.2 to 1.8	2.1	1.7 to 2.6	3.6	2.8 to 4.6
SSP5-8.5	1.6	1.3 to 1.9	2.4	1.9 to 3.0	4.4	3.3 to 5.7

High end pathways, and SSP5-8.5 particularly, are often taken to represent business as usual (BAU), that is, the future course of greenhouse gas emissions this century if no effective mitigation efforts are taken. However, there is an ongoing debate about which emission pathways should be associated with BAU (Hausfather and Peters, 2020; Schwalm et al., 2020a, 2020b). An emission pathway like SSP5-8.5 combines a socioeconomic story relating to future energy use with a trajectory of atmospheric greenhouse gas concentrations that story could explain. The story is the shared socioeconomic pathway, or SSP, while the trajectory is called a representative concentration pathway, or RCP. RCPs are associated with numbers that denote the radiative forcing they are expected to generate in 2100. Thus, in the year 2100, RCP8.5 would yield 8.5 watts of radiative forcing per meter squared. SSP5-8.5, then, conjoins the fifth socioeconomic pathway considered by the IPCC, SSP5, with RCP8.5. Critics of RCP8.5 as a BAU trajectory argue that SSP5 assumes an improbable increase of coal combustion throughout the remainder of this century. Those who defend RCP8.5 as a BAU trajectory claim that it could arise from scenarios with less future coal use that take into account effects of deforestation and climate feedbacks, and moreover that RCP8.5 remains close to measured atmospheric greenhouse gas concentrations. We make no attempt here to resolve this disagreement. Even if critics are correct that RCP8.5 is an improbable upper end trajectory, the fact remains that +4°C falls within the highly likely range by 2100 for less extreme BAU scenarios like SSP3-7.0. Together with the assumption that +4°C is compatible with global collapse scenarios, that is enough to suggest that the risk of global collapse this century should be taken seriously. It follows that mitigation which lessens or avoids risks of +4°C is mitigation that protects from the risk of global collapse. In short, this kind of mitigation would have significant importance to those who might expect to live to the end of the century, since this mitigation would avert the risks of extremely dangerous outcomes in this timeframe (we expand on these points in the next section).

There are even more plausible scenarios whereby climate change can lead to local collapses or the broken world. Low lying islands or coastal areas may be abandoned due to sea level rise and arid regions might become uninhabitable due to extreme heat and drought (Carleton and Hsiang, 2016; Mazo, 2010). Philosophers have considered the moral obligations that such local collapses would create (Capisani, 2020; Draper, 2023; Risse, 2009; Wündisch, 2019). Importantly for the present purposes, local collapses become a serious concern at levels of global heating well below +4°C. Consider, for example, SSP2-4.5, which the IPCC characterizes as an intermediate scenario in which some mitigation efforts are taken. The very likely range for 2081–2100 given SSP2-4.5 is +2.1°C to +3.5°C, with a best estimate of +2.7°C (IPCC, 2021, 14). While less dire than SSP5-8.5 or SSP3-7.0, global heating above +2°C as expected under SSP2-4.5 would put coral atolls, such as the Maldives and Kiribati, at risk of becoming uninhabitable due to frequent wave-driven flooding that spoils freshwater aquifers (Storlazzi et al., 2018). In fact, the very likely temperature range for SSP1-2.6 by 2081–2100 is +1.3°C to +2.4°C, with +1.8°C as the best estimate. SSP1-2.6 is one of two pathways the IPCC describes as having “very low and low GHG emissions and CO_2_ emissions declining to net zero around or after 2050, followed by varying levels of net negative CO_2_ emissions” (IPCC, 2021: fn 25). Thus, local collapses remain a concern even under relatively optimistic scenarios.

Heating above +3°C, which falls within SSP2-4.5's very likely range for 2081–2100, would threaten the habitability of tropical and subtropical regions across the globe (Andrews et al., 2018; Im et al., 2017; Rohat et al., 2019) as well as risk drought and declines in food production in temperate climates such as North America and Europe (Samaniego et al., 2018; Schlenker and Roberts, 2009). Under such conditions, the broken world's scenario of widespread but not global collapses combined with general food and water scarcity does not seem farfetched. In sum, while global heating of +4°C might be viewed as a rough threshold for global climate collapse to be a significant concern, a case can be made for thinking that serious risks of local collapses and the broken world arise at lower temperature increases.

We believe taking the risk of climate collapse seriously has important consequences for the ethics of climate change. For instance, consider Posner and Weisbach's (2010) proposal that only the feasible climate treaty may be one in which more vulnerable nations compensate less vulnerable for their emission reductions. This claim is based on the idea that some countries (e.g., Bangladesh) are severely threatened by climate change while others (e.g., the United States) are not, and furthermore that “international Paretianism,” in which an agreement benefits some countries and is not contrary to the interests of any, is a constraint on feasibility. While a number of philosophers have sharply criticized international Paretianism as a desideratum of climate agreements (Bernstein, 2016; Broome, 2012; Budolfson, 2021; Frisch, 2012; Steele, 2021), critics rarely challenge the premise that climate change poses little threat to the capacities and existence of the less vulnerable governments. However, such an assumption is plainly called into question by the above discussion: high emission scenarios threaten global climate collapse, while the intermediate scenario risks a broken world where currently wealthy and less vulnerable countries might survive only in a weakened and impoverished form. In these contexts, it does not matter what ideal policy would look like, since there might not be a government capable of enacting it.

In light of these various considerations, we have a simple conclusion. The science reviewed in the AR6 shows that climate collapse should no longer be viewed as a risk only for future generations or especially vulnerable people and places.

## And now for the good news

In this section, we discuss two more hopeful recent developments. First, more recent climate science suggests that climatic effects of mitigation will be discernable more quickly than previously thought. Second, costs of renewable energy have fallen significantly in the past decade, largely as the result of an international process whereby policies that promote uptake of renewables create economic incentives for innovations and efficiencies that lower prices. Taken together, these developments entail that both current and future generations will enjoy significant climatic benefits of mitigation, and that mitigation policies adopted in one place can increase the probability of mitigation elsewhere. Both of these consequences conflict with key assumptions found in some prominent framings of mitigation.

### Faster mitigation

It is sometimes suggested that due to the long atmospheric half-life of CO_2_ (approximately 120 years) and inertia in processes related to climate change, such glacial melt and sea level rise, benefits of reductions of greenhouse gas emissions are mostly deferred to the future (Moellendorf, 2015: 174). By the time the benefits of mitigation kick in, the reasoning goes, currently living people will mostly be dead and gone, so the benefits will largely accrue to future generations. Such ideas underlie what Gardiner calls “intergenerational buck passing,” in which each generation prefers to continue emitting and pass the costs of mitigation along to future generations (Gardiner, 2011, 32–38). Like Gardiner, Mulgan also introduces a model of the ethics of mitigation that assumes current generations do not benefit from climate change mitigation (Mulgan, 2018: 535).

Yet the belief that past emissions make future warming unavoidable has been recognized as a mistake within climate science for over a decade (Matthews and Solomon, 2013; Matthews and Weaver, 2010). In a 2010 letter published in *Nature Geoscience*, Matthews and Weaver explain that the term “committed warming” has often been misunderstood to mean warming that is inevitable or impossible to prevent. Instead, committed warming refers to the equilibrium mean global warming that would result from maintaining greenhouse gas concentrations at current, heightened levels. However, committed warming is not inevitable, because atmospheric greenhouse gas concentrations would decline if emissions ceased. Moreover, human actions and policies affect how quickly greenhouse gases are removed from the atmosphere, through enhancing carbon sinks (e.g., by afforestation) and implementation of technologies like carbon dioxide removal (Mintz-Woo and Lane, 2021). The relevance of human decisions to the rate of decline in atmospheric greenhouse gas concentrations is reflected in the IPCC's emission scenarios.

Moreover, the Synthesis Report of the AR6 states with high confidence that “Deep, rapid, and sustained reductions in greenhouse gas emissions would lead to a discernible slowdown in global warming within around two decades, and also to discernible changes in atmospheric composition within a few years.” This statement is based upon more recent modeling research that improved upon earlier work. Prior modeling had suggested that aggressive mitigation would create a “heat pulse” due to the reduction of sunlight-reflecting aerosols generated by fossil fuel combustion; but a *Nature* study showed that, within the IPCC's low-emission pathways, no heat pulse occurs and global mean temperatures begin to fall within two decades (Shindell and Smith, 2019). The supposed heat pulse, it turned out, was an artifact of modeling assumption in which mitigation consisted of an immediate cessation of emissions, rather than a graduated phaseout.

The points made in the previous paragraph are reflected in the IPCC temperature projections given in Table 1 indicates that mitigation, understood as following one of the IPCC's low-emission scenarios, matters enormously to the well-being of current generations (all currently living people).^4^ Start by considering the IPCC's long term (2081–2100) projections in connection with climate collapse. The probability of exceeding or approaching the +4°C threshold by the last two decades of this century is much higher in the AR6's two high than in its low emission pathways. If +4°C is taken as a rough proxy for risk of global collapse, this means that aggressive climate change mitigation stands to significantly reduce that risk for *billions of currently existing people*.^5^ According to some estimates, two-thirds of infants born today may live to see the end of the twenty-first century.^6^ Similarly, a significant portion of those currently aged less than 20 will live to 2080. And note that global collapse is not just intrinsically objectionable, it would also undermine many other goals that people have (i.e., a stable society is a precondition for many, if not all, conceptions of the good life). If so, global collapse would undermine many types of lives, and risk of global collapse would be a very serious threat to anyone facing it. In short, younger people clearly stand to benefit from mitigation.

Mitigation would have significant benefits for many current adults as well as young people. People who are middle aged today are likely to live to mid-century, when climatic differences between high and low emission scenarios become significant. Older adults are especially susceptible to some climate impacts, like heatwaves, and often suffer from medical conditions that make it difficult to evacuate when extreme weather threatens. Thus, a world where global mean warming at midcentury is 1.6°C will be much more hospitable to people in their 70s and 80s (i.e., people in their 40s to 50s in the 2020s) than a world where global warming has reached 2.4°C. For adults living in highly climate vulnerable places, like small island states, such a difference may be crucial for the continued survival of their communities. If local collapse is reasonably thought of as catastrophic for those who are exposed to it, there are adults alive today who would benefit from reducing that catastrophic risk.

Moreover, we would argue that reducing the risk of climate collapse this century could even be a significant benefit for adults residing in places that are unlikely to collapse in their own lifetimes. Scheffler (2013, 2018) argues that the belief in a posthumous future with which one shares personal, cultural or other connections is of fundamental importance to most people. People want their children and grandchildren to have good lives that extend beyond their own, find deep satisfaction in seeing their cultural traditions carried on by younger generations, and it is often important for scholars, artists, and social reformers to see themselves as contributing to worthwhile multi-generational endeavors. As these examples suggest, a sense of meaning in life is often bound up with the expectation of being connected to a future that will continue after one has passed from the scene. Given this, there are potential harms to current (older) people who may foresee that climate change threatens civilization collapse. Such a collapse would threaten to sever many of the ties that people desire to have with the future. Conversely, it could be a psychologically and morally relevant benefit to current (older) people to know that these ties were *not* threatened by civilization collapse. Scheffler's claims about the importance of connections to a future after one's death, therefore, suggests that climate collapse would be devasting not merely to those who that endured it, but also to those who could see it coming. Avoiding these threats could thus be deeply reassuring and beneficial, even to those who might not directly experience the worst climate effects.

Finally, while the climatic impacts of climate change mitigation are likely to be fairly modest within the next two decades, some significant co-benefits of mitigation are immediate. Perhaps the most important of these is reduced fine particulate air pollution (Shindell et al., 2021). Recent estimates of worldwide premature mortality from fossil fuel generated particulate air pollution range from 3.6 to 8.7 million annually (Lelieveld et al., 2019; Vohra et al., 2021). And other co-benefits of a transition away from fossil fuels exist, such as increased energy security and reduced environmental damage linked to fossil fuel transport and extraction (Karlsson et al., 2020; Scovronick et al., 2019; Watts et al., 2021). Moreover, given declining costs of renewable energy, an earlier transition from fossil fuels may reap significant cost savings in coming decades (Way et al., 2022). Indeed, some have argued that co-benefits alone suffice for a strong cost-benefit argument for transitioning away from fossil fuels (Green, 2015). Moreover, according to the AR6, “The global economic benefit of limiting warming to 2°C is reported to exceed the cost of mitigation in most of the assessed literature” (IPCC, 2022: C.12). Moreover, the IPCC states that models find that mitigation has favorable margins of benefits to costs *within this century* unless they either assume unusually low climate damages or employ a high discount rate (IPCC, 2022: C.12.3).

Mitigation, then, offers clear benefits to current generations, and the above discussion suggests that the case for net benefits of mitigation to currently living people is strong. Nevertheless, the contrary assumption—that mitigation is a net cost for current generations—remains influential. As a prominent example, consider Gardiner's (2011) “intergenerational buck passing” model, which is intended as a simplified representation of the core moral challenge posed by climate change.^7^ Gardiner proposes that intergenerational buck passing is rooted in the simplified “pure intergenerational problem” (36), which is characterized by the following two claims:

(PIP1) Almost every generation prefers the outcome produced by everyone restricting its pollution over the outcome produced by everyone overpolluting.

(PIP 2) When each generation has the power to decide whether or not it will overpollute, each generation (rationally) prefers to do so, whatever the others do.

Gardiner (2011: 37) explains that the first generation is the chief exception covered by the “almost” in (PIP1): the first generation in the sequence receives no benefits from the mitigation of others, and so is indifferent to their emissions.

According to Gardiner's (explicitly simplified) model, then, mitigation will occur only if some generations altruistically choose to sacrifice their own interests for the sake of the future, and the challenge is to convince current generations of the moral obligation to do just this. In a recent book, Shue (2022: 10) similarly frames the ethics of climate change mitigation around providing a compelling answer to the question: “Isn’t the burden of promptly and firmly initiating a global Energy Revolution too heavy for the current generation alone to be expected to bear?”

But given risks of climate collapse this century along with the potential for renewables to generate public health co-benefits and cost-savings (which, admittedly, also include future savings), why should one assume that mitigation is a net cost for current generations? Why assume, in short, that mitigation is a sacrifice? In fact, Gardiner (2011) considers the argument that mitigation can benefit current generations by preventing catastrophic climate impacts and gives two responses to it: first, that a variety of cognitive biases cause people to discount or dismiss risks posed by climate change, and second, that members of current generations would experience benefits of mitigation only at the ends of their lives. Since these are direct responses to our claim that mitigation stands to benefit current generations, they deserve careful consideration.

Gardiner (2011: 193–196) describes biases of risk perception that can cause people to disregard or discount risks that are improbable or have not impacted them personally and suggests that these pose an obstacle to action on climate change. While Gardiner is surely right that cognitive biases influence people's reactions to climate change, this does not support intergenerational buck passing as an adequate model for climate ethics. Intergenerational buck passing claims that mitigation—as a matter of fact, not perception—is a net cost for current generations, and this assumption is called into question by the observation that preventing climate collapse would benefit currently existing people (e.g., by avoiding the extremely dangerous end-of-century outcomes associated with the scenarios without mitigation).

Moreover, it is questionable whether biased risk perception can explain inaction on climate change (Atkinson and Jacquet, 2022). The effects of chlorofluorocarbons on stratospheric ozone are remote from most people's everyday lives, yet this did not prevent the Montreal Protocol on Substances that Deplete the Ozone Layer. So, inaccessibility from ordinary experience is not, by itself, sufficient to explain inaction on environmental issues. And in any case, climate change is no longer an improbable or psychologically distant threat. Severe climate impacts, such as extreme heatwaves, droughts, forest fires, flooding, and coastal erosion, and their potential to disrupt daily life are becoming increasingly obvious to people around the globe. Survey work suggests that concern about climate change is rising across all age groups (Milfont et al., 2021), and a growing number of younger people suffer from climate anxiety (Wu et al., 2020).

Gardiner's other main response is that current generations would only benefit from mitigation at the tail end of their lives, so it is in their self-interests to continue business as usual and invest in adaptation measures that will hold out for a few decades (2011: 200). Gardiner also suggests that the relevant sense of “current generation” is the age group whose members have the power to make decisions, which he associates with people aged 45 to 65 (2011: 173–174). Defining “current generation” in this way would circumvent claims, like those made above, that people under twenty would benefit enormously from climate change mitigation in the latter half of this century (Thiery et al., 2021). Gardiner also suggests that those in power are often beset by incentives, such as the need to win upcoming elections, that make them focus on immediate rather than long-term problems (2011: 174).

However, Gardiner's second response also fails as a defense of the assumption that current generations do not significantly benefit from mitigation. As explained above, aggressive mitigation will have significant benefits for adults and well as younger people, and it is unclear why these benefits should be of scant concern to decision-makers focused on nearer-term problems. In addition, we do not agree that the current generation should be defined in connection with those who currently hold power. Among people aged 45 to 65, there are vast inequalities in who has power to make decisions about climate change policy, and it is not reasonable to assume that the short-term interests of political elites are identical to the interests of a human generation. If power to make decisions is emphasized, the focus should not be on generations but on the disproportionate political influence of extremely wealthy people, fossil fuel corporations, and the like. That certain powerful individuals and organizations have a vested interest in opposing climate change mitigation is a very different proposition than the assumption that mitigation only benefits future generations.^8^

### Social tipping dynamics

Since 2000, photovoltaic solar electricity generation has gone from an expensive niche electricity source to being the cheapest means of electricity generation (Nemet, 2019). Declining cost curves for wind and lithium-ion batteries have also occurred (Way et al., 2022). These developments are relevant to our discussion in two main ways. First, declining costs of renewable energy support the claim made in 4.1 that aggressive mitigation stands to benefit current people. As AR6 reports: “Feasible, effective, and low-cost options for mitigation and adaptation are already available, with differences across systems and regions (*high confidence*).” Second, and specifically relevant for this subsection, *how* costs of renewable energy technologies have declined indicates the potential for positive feedback mechanisms relating to mitigation, that is, mechanisms whereby mitigation by some makes others more likely to mitigate, which in turn makes still others more likely to mitigate, and so on. Such mechanisms are often called *social tipping dynamics*, since action by one breaches social tipping points by others (sometimes with domino effects) (cf. Otto et al., 2020; Sharpe and Lenton, 2021; Winkelmann et al., 2022).

Consider feed-in tariffs implemented by Germany in 2000 to promote renewable electricity generation (Nemet, 2019). At the time, wind and solar power were significantly more expensive than the primary fossil fuel alternatives, coal and gas. Consequently, Germany implemented a program in which public utilities paid above-market prices for electricity generated from renewable sources. The resulting increased demand for solar and wind electricity generation stimulated manufacturing of these technologies, which ultimately led to improved workforce skills, economies of scale, and complementary technical innovations that brought down prices. This process spread internationally, and China eventually emerged as the world's largest producer of solar panels (also with significant governmental support). By the mid-2010s, solar electricity generation was able to outcompete coal and gas strictly on price in many parts of the world. National feed-in tariffs, then, were an important contributor to reducing prices of solar electricity generation, which in turn contributed to worldwide increases in the uptake of renewables.

Sharpe and Lenton (2021) explain that tipping points in complex systems can lead to policies whose effects are disproportionate (e.g., feed-in tariffs in Germany sparking a worldwide solar and wind boom) and involve self-reinforcing feedback loops (e.g., rising production of solar panels leads to lower prices, which increases demand, which leads to more production, etc.). They suggest that climate change policy should identify such tipping points, and craft interventions aimed at breaching them. For electricity generation, Sharpe and Lenton identify two tipping points besides renewable power beating fossil fuels on price: renewable finance being cheaper than fossil fuel finance, and building new renewable electricity generation becoming cheaper than running existing fossil fuel plants. By 2023, the second of these tipping points appears to have been crossed for nearly all coal fired plants within the United States, thanks in part to renewable energy incentives in the 2022 Inflation Reduction Act.^9^ However, financing for renewable energy remains a major challenge in developing countries (International Energy Agency, 2021).

The dramatic declines in costs of renewable electricity generation in recent decades, then, is an important example of a social tipping dynamic that is well underway but still in progress. Instigating or accelerating social tipping dynamics in many economic sectors, including road transport, shipping, air travel, and industrial processes like steel and concrete manufacturing, is, therefore, a promising approach to mitigation. We claim that social tipping dynamics strongly challenge the idea that decisions about mitigation can be modeled as an *n*-person prisoner's dilemma, or “tragedy of the commons.”

An *n*-person prisoner's dilemma is defined by two conditions: (1) everyone is better off when all cooperate than when all defect, and (2) each agent is better off defecting no matter what the other agents do. To make this concrete, imagine a game where 10 players decide whether to contribute money to a pot under the condition that the total will be multiplied by 5 and then evenly divided among all 10. For simplicity, suppose each player can contribute $1 or nothing. How much should each player contribute if their only aim is to maximize their payoff? If all contribute $1, then each receives a payoff of $5 for a net profit of $4. Of course, if none contribute, all walk away with no profit at all. So, the first condition of an *n*-person prisoner's dilemma is met: all are better off if all cooperate than if all defect. But consider the payout that each player receives from their own contribution: $1 × 5 ÷ 10 = $0.5. So, no matter what the others do, each player loses 50 cents by contributing. The second condition of an *n*-person prisoner's dilemma, therefore, is also satisfied in this example.

The *n*-person prisoner's dilemma is often considered as a model for climate change (e.g., Steele, 2021). Avoiding catastrophic climate change requires mitigation by governments around the world, where costs of mitigation are borne individually while the climatic benefits are distributed regardless of individual contributions. Thus, the situation appears to resemble the example from the foregoing paragraph. If everyone mitigates, climate collapse is avoided and all are better off than if none mitigated. However, since costs of mitigation accrue individually while benefits disperse collectively, each actor might be better off not mitigating no matter what the others do.

There are well-known ways that mitigation might not be an *n*-person prisoner's dilemma. For example, suppose there are thresholds beyond which catastrophe will occur, say, global mean warming above 2°C. Moreover, mitigation by some large emitters, like China and the United States, is necessary to avoid crossing the threshold. Consequently, if a large emitter were confident that a sufficient number of other actors would mitigate, then it would be in their interests to mitigate as well, because their actions would prevent catastrophe. Such situations are known as coordination games. In a coordination game, it is in the self-interest of some actors to cooperate provided that a sufficient number of others do, so that the second condition of a prisoner's dilemma is not satisfied. Coordination games have been studied in experiments with human subjects, and findings suggest that cooperation is likely, especially if players are able to communicate and observe the behaviors of others (Barrett, 2011; Tavoni et al., 2011).^10^

Unfortunately, cooperation in coordination games is sensitive to uncertainty about the location of the threshold. Barrett and Dannenberg (2012) show that if actors are uncertain where this threshold lies and what level of emissions may trigger it, then a coordination game reverts to an *n*-person's prisoner's dilemma. Moreover, they report experimental results showing that people are unlikely to cooperate in coordination games where thresholds are uncertain. Some recent philosophical work takes Barrett and Dannenberg's results as a rationale for modeling mitigation as an *n*-person prisoner's dilemma (Steele, 2021).

However, Barrett and Dannenberg's (2012) work on uncertainty in coordination games predates the emergence of renewables as the cheapest forms of electricity generation, a fact reflected in their modeling assumptions. Two assumptions in particular matter here. First, they assume that mitigation is always costly. This assumption is increasingly questionable in a world where it is cheaper to generate electricity from photovoltaic solar than fossil fuels (Way et al., 2022). Second, Barrett and Dannenberg's model assumes that costs of mitigation are independent of mitigation undertaken by others. This assumption is again no longer reasonable given documented social tipping dynamics, such as that sparked by Germany's 2000 feed-in tariffs (Nemet, 2019). Consider a simple model to clarify this point.

Imagine a game where 10 players contribute money to a pot that will be multiplied by 5 and then divided equally among them. But this time, players contribute in order and each contribution generates a 25-cent rebate for all ensuing contributors. Thus, the first contributor puts in $1, and receives 10% of the common pot. The second contributor receives a 25-cent rebate, so has contributed $1 to the common pot at a cost of only 75 cents; the third contributor receives a 50-cent rebate, and so on. The cost for the fifth contributor would be zero, and costs would be negative for all contributors after that. Call this a *tipping game* (Barrett and Dannenberg, 2017). The declining costs of cooperation in a tipping game are intended to represent the declining costs of renewable energy technologies resulting from increased production stimulated by investments and policies of a relatively small number of leading actors. In some cases, negative costs in a tipping game could occur, corresponding to cases where renewables are cheaper than fossil fuels. The defining feature of a tipping game is that each cooperative action reduces the costs of cooperation for subsequent actors.

In a tipping game, cooperation can be in an individual's self-interest. This is most obviously the case when prior cooperation is sufficient to make the cost of further cooperation negative. However, some agents may be better off cooperating in a tipping game even before this tipping point is reached. Return to the example from the previous paragraph. Suppose that none have yet contributed, but player 1 knows that player 2 will contribute if the cost is less than $1, that players 3 and 4 will contribute if the cost is 50 cents or less, and that everyone else will contribute if the cost is zero or less. Then player 1 gains $4 by contributing. Of course, the actual circumstances of climate change mitigation are far more complex than this toy game. Our point here is only that social tipping dynamics undercut the prisoner's dilemma as a model for climate change.

## Reframing climate ethics

A framing describes the central aspects of an issue and suggests key unresolved questions. For example, Posner and Weisbach (2010) assume that mitigation is not in the self-interests of less climate-vulnerable countries and that only climate treaties consistent with international Paretianism are feasible. Given this set up, the key question is how to induce the non-vulnerable countries to agree to emission reductions. Gardiner's (2011) intergenerational buck passing model is a framing in which the crucial premise is that climatic effects of emissions are “backloaded” so that the benefits of mitigation are deferred to the future. Given this framing, the crucial question is how to convince current people of the moral necessity to make sacrifices for future generations. Finally, framing climate change as a prisoner's dilemma entails that mitigation is collectively beneficial but individually costly, and thus focuses attention on the question of how to prevent free riding. Despite their differences, each of these framings characterizes mitigation as a sacrifice, and the differences mainly have to do with who would sacrifice their self-interests: wealthy countries, current generations, or each individual.

Yet these framings are increasingly questionable given the state of current climate science and renewable energy technologies. The assumption that some countries do not face catastrophic risks from climate change is no longer tenable, nor is the notion that mitigation only benefits future generations, and falling prices of renewables undermine the key assumption of the prisoner's dilemma that mitigation is always individually costly. Therefore, we suggest an alternative framing that reverses each of these problematic assumptions: (1) climate collapse is a serious global risk, not merely a concern for especially vulnerable places; but (2) mitigation stands to substantially benefit current as well as future generations, and (3) social tipping dynamics and ensuing reduced costs of renewable energy present opportunities for decarbonization of the economy with direct economic benefits. Given these premises, the central question is how to fairly and efficiently accelerate social tipping dynamics so that the decarbonization of the economy happens quickly enough to avert catastrophic climate change.

Some clarifications of these three claims may be helpful. The first states that the risk of climate collapse is non-negligible for all but recognizes differences in climate vulnerability and the imminence and probability of climate collapse risks. That catastrophic climate impacts are a worldwide risk is compatible with those risks being more severe in some places than others. The second claim asserts a collective, net benefit for current generations from mitigation, and is consistent with mitigation benefiting some more than others and with some, especially vested fossil fuel interests, suffering losses. Finally, the phrase “direct economic benefits” in claim (3) refers to cost savings due to replacing fossil fuels with renewables. Thus, direct economic benefits do not include reduced climate risks and co-benefits like improved air quality. Moreover, we believe that the social tipping dynamics mentioned in (3) can occur in many different sectors of the economy, including electricity generation, light road transport, shipping, air travel, agriculture, and industrial processes like steel and concrete manufacturing. In some of these sectors, such as electricity generation, key tipping points are approaching or already in the rear-view mirror. In other cases, such as decarbonizing steel production, we are in much earlier stages (Sharpe, 2023). But our suggestion is that each of these cases is better viewed from the perspective of a tipping game—in which actions of earlier mitigators reduce costs for later mitigators—than an *n*-person prisoner's dilemma in which mitigation is contrary to each actor's self-interests no matter what the others do.

Consider the key ethical questions that ensue from our framing. Since mitigation, according to our approach, stands to generate substantial economic benefits, the distribution of those benefits is a concern from the perspective of justice. From an international perspective, justice would plausibly demand that wealthier nations have an obligation to ensure that these benefits are shared with lower-income countries. Financing for renewable energy projects in developing countries is a good example of this point (International Energy Agency, 2021). Such financing will help build infrastructure that generates electricity at lower cost and with less local air pollution, thereby providing long lasting benefits in places where they are sorely needed.^11^ Moreover, given that climate collapse is a global risk, it is the interests of wealthier countries that expanded electricity access in the developing world be powered by renewables rather than fossil fuels.

Treating tipping games as a model for mitigation also raises interesting strategic questions. Later contributors in a tipping game may profit more than earlier contributors. Moreover, early contributors risk losses if it is uncertain whether their actions will generate a social tipping dynamic, for example, if a country invests heavily in an ultimately unsuccessful zero-emission steel manufacturing technology. These considerations suggest that collective benefits may not be attained in a tipping game as a result of too many actors waiting for others to contribute before doing so themselves. While such scenarios are possible, we suggest that they are less worrisome than free-riding in a prisoner's dilemma. First, collective benefits in a tipping game can result from the leadership of relatively few actors who set in motion a positive feedback loop that eventually makes cooperation the self-interestedly rational option for all (Sharpe, 2023). So, compared to a prisoner's dilemma, broad cooperation in a tipping game takes fewer cooperators to get rolling and is self-reinforcing once established. Second, waiting in a tipping game can also be risky. For example, an auto manufacturer that postpones shifting its business to electric vehicles risks being unable to maintain market share if electric vehicles win out. Governments, moreover, can enact policies that reduce risks of leading (e.g., tax rebates for electric vehicles) and increase risks of waiting (e.g., mandating zero-emission vehicles by a future date).

## Conclusion

We have argued that the current state of climate science and renewable energy technologies should prompt a reframing of debates about climate change. According to the framing, we propose: (1) climate collapse is a global risk, but (2) mitigation will substantially benefit current generations, and (3) social tipping dynamics and falling costs of renewable energy suggest that mitigation can generate direct economic benefits. Such a framing is at once frightening—because it emphasizes how close the world stands to the precipice—and hopeful—because it suggests that solutions are already emerging and that there is a recipe for accelerating them. Our framing differs fundamentally from more familiar alternatives in which mitigation is depicted as a self-sacrifice.

But why do framings matter? We think one pragmatic reason to consider them is that they motivate action. Prinzing (2023) argues that self-sacrifice framings are ineffectual motivators. In a similar vein, Atkinson and Jacquet (2022) criticize claims that humans are inherently subject to psychological tendencies or biases (e.g., putting self-interest before the common good, prioritizing immediate over long term concerns, etc.) that make it difficult for them to effectively respond to climate change. They argue that such claims oversimplify what is known about human psychology and rationalize inaction. By avoiding the problematic features noted by these authors, we hope that our framing can more effectively motivate action on climate change.

## Notes

1. Transcript of the speech by Sir David Attenborough COP24, Katowice, Poland, 3rd December 2018: https://unfccc.int/sites/default/files/resource/The%20People%27s%20Address%202.11.18_FINAL.pdf

2. We remain neutral on the correct theory of well-being. See Fletcher (2016) for an overview of leading approaches.

3. Global collapse, and possibly the broken world, could be viewed as a “global catastrophic risk” (cf. Beard et al., 2021).

4. Shue (2022: 10) also defines “current generation” in this manner.

5. This is a back of the envelope estimate given that there are approximately two billion children between the ages of 0 and 14 alive today.

6. For example, see https://www.brookings.edu/blog/future-development/2020/01/14/living-into-the-22nd-century/.

7. For instance, Moellendorf (2022: 124) describes this, we believe accurately, as “an influential explanation of the problems of achieving robust mitigation.”

8. Gardiner (2011: 64–68) does, in fact, grant that climate change mitigation might only be contrary to vested fossil fuel interests but suggests that intergenerational buck passing remains a viable model, nevertheless. We disagree. Gardiner's intergenerational buck passing model is founded on supposed conflicting interests between current and future generations. In contrast, an emphasis on vested fossil fuel interests implies conflicting interests among people living at the same time who differ in their economic dependence on fossil fuel extraction.

9. See: https://financialpost.com/pmn/business-pmn/replacing-us-coal-plants-with-solar-and-wind-is-cheaper-than-running-them

10. There are interesting and independent arguments that international climate negotiations should not be modeled as a collective action game (Aklin and Mildenberger, 2020), but we can remain agnostic with respect to these for the purposes of our arguments.

11. Shue (2022: 66−67) also discusses obligations high-income countries to support low-income countries in obtaining benefits of renewable electrification.
